# Development and Evaluation of a Barley 50k iSelect SNP Array

**DOI:** 10.3389/fpls.2017.01792

**Published:** 2017-10-17

**Authors:** Micha M. Bayer, Paulo Rapazote-Flores, Martin Ganal, Pete E. Hedley, Malcolm Macaulay, Jörg Plieske, Luke Ramsay, Joanne Russell, Paul D. Shaw, William Thomas, Robbie Waugh

**Affiliations:** ^1^The James Hutton Institute, Dundee, United Kingdom; ^2^TraitGenetics GmbH, Gatersleben, Germany

**Keywords:** barley, SNP, genotyping chip, iSelect, exome capture

## Abstract

High-throughput genotyping arrays continue to be an attractive, cost-effective alternative to sequencing based approaches. We have developed a new 50k Illumina Infinium iSelect genotyping array for barley, a cereal crop species of major international importance. The majority of SNPs on the array have been extracted from variants called in exome capture data of a wide range of European barley germplasm. We used the recently published barley pseudomolecule assembly to map the exome capture data, which allowed us to generate markers with accurate physical positions and detailed gene annotation. Markers from an existing and widely used barley 9k Infinium iSelect array were carried over onto the 50k chip for backward compatibility. The array design featured 49,267 SNP markers that converted into 44,040 working assays, of which 43,461 were scorable in GenomeStudio. Of the working assays, 6,251 are from the 9k iSelect platform. We validated the SNPs by comparing the genotype calls from the new array to legacy datasets. Rates of agreement averaged 98.1 and 93.9% respectively for the legacy 9k iSelect SNP set (Comadran et al., 2012) and the exome capture SNPs. To test the utility of the 50k chip for genetic mapping, we genotyped a segregating population derived from a Golden Promise × Morex cross (Liu et al., 2014) and mapped over 14,000 SNPs to genetic positions which showed a near exact correspondence to their known physical positions. Manual adjustment of the cluster files used by the interpreting software for genotype scoring improved results substantially, but migration of cluster files between sites led to a deterioration of results, suggesting that local adjustment of cluster files is required on a site-per-site basis. Information relating to the markers on the chip is available online at https://ics.hutton.ac.uk/50k.

## Introduction

Barley (*Hordeum vulgare* L.) is a cereal crop of major importance, ranked fourth grain crop in the world by the UN's Food and Agriculture Organization in terms of production volume (http://faostat.fao.org). Its major uses are as animal feed, for brewing and distilling, and to a minor extent, human nutrition.

Molecular markers are now widely utilized in the breeding of new varieties of crop plants, ranging from dense genome-wide marker analyses to characterize parental lines, through to one or a few markers tagging specific traits in segregating populations. Single nucleotide polymorphisms (SNPs) are today's marker of choice due to their abundance, relative ease of discovery in high-throughput sequencing data (Kumar et al., 2012) and costs per data point. The design of SNP genotyping arrays for germplasm characterization has become commonplace, and many important crop plant species now have their own custom SNP genotyping arrays, for example wheat (Winfield et al., 2016), rice (Chen et al., 2014), maize (Ganal et al., 2011), potato (Vos et al., 2015), rapeseed (Clarke et al., 2016; Mason et al., 2017), apple (Bianco et al., 2014, 2016), tomato (Sim et al., 2012), and others reviewed in Ganal et al. (2012).

High throughput genotyping in barley was first introduced in 2006 with the development of two Illumina GoldenGate assays (Fan et al., 2003) that featured 1,572 SNP markers each (Close et al., 2009). A new genotyping platform followed in 2009 that introduced larger numbers of markers based on SNP discovery in Next Generation Sequencing data. The 9k Illumina Infinium iSelect Custom Genotyping BeadChip (Comadran et al., 2012) included 2,832 markers developed for the previous GoldenGate assays and 5,010 additional markers based on SNP marker discovery in Illumina RNAseq data from 10 UK elite cultivars. The iSelect technology currently offers the potential of up to 700k custom targets (SNPs, indels, and CNVs) per array and 24 samples can be processed at a time per chip (https://www.illumina.com/content/dam/illumina-marketing/documents/products/technotes/technote_iselect_design.pdf).

With the continuing drop in sequencing costs, it has become cheaper and easier to identify new markers in ever increasing numbers. This prompted us to develop a new genotyping platform which provides more in-depth coverage of the barley genome. Publication of a new barley genome assembly (Beier et al., 2017; Mascher et al., 2017) that explores the complex 5.1 Gbp genome at an unprecedented level of resolution has established a new era for barley genetics. The availability of a chromosome-scale assembly with detailed gene annotation means that markers can be placed accurately and interpreted in the context of associated genes.

Here, we report the development and evaluation of a new 50k Illumina Infinium iSelect SNP genotyping array that includes most of the markers from the previous barley genotyping platforms and features 42,316 new SNPs derived from exome capture data of 170 carefully selected barley accessions. We chose this number of markers as it provides a favourable tradeoff between genotyping costs and marker density. Marker discovery was based on the 2017 barley genome assembly (Beier et al., 2017; Mascher et al., 2017) that provides accurate placement of the markers and the genes from which they were derived on the physical map.

## Materials and methods

### Germplasm selection

**Figure 1:**
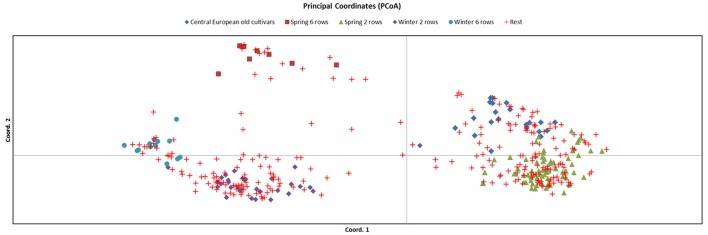
Principal Coordinate Analysis (PCoA) of 394 accessions genotyped with 6,917 polymorphic SNPs. The core set of 148 barley lines chosen are classified into 5 groups: (i) Central European old cultivars; (ii) spring 6 row; (iii) spring 2 row; (iv) winter 2 row and; (v) winter 6 row, and the remaining 246 accessions (classified as “rest”) are shown.

To select the most representative set of germplasm we used a combination of approaches and available datasets. First, we identified a set of 394 cultivated accessions for which we had published SNP genotypic information and which were part of previous studies to examine genetic diversity in cultivated barley (Rostoks et al., 2005; Cockram et al., 2010; Comadran et al., 2012). The genetic relationship between these accessions was visualized by generating a Principal Coordinate Analysis (PCoA) using GenAlEx 6.5 (Peakall and Smouse, 2012) for 6,917 polymorphic SNPs (Figure 1). In this set we categorised each accession into different groups based on growth habit, row type, date of introduction and origin, where possible. From this we identified a set of 148 accessions representing most of the variation and allelic diversity (Figure 1 groups highlighted as: central European old cultivars; spring 6 row; spring 2 row; winter 2 row and winter 6 row). This was supplemented with a further 22 landraces sourced from the Germplasm Resources Unit (GRU) at the John Innes Institute and Science and Advice for Scottish Agriculture (SASA), for which no SNP data was available for comparison. This resulted in a total number of 170 lines for exome capture.

### SNP discovery

Exome capture (EC) sequence data (Mascher et al., 2013) from these 170 accessions was made available prior to publication for the purposes of SNP discovery and iSelect development (Waugh et al., unpublished data). Illumina read processing and variant calling was carried out in line with the recommendations made in the Genome Analysis Toolkit (GATK) Best Practices documentation (Van der Auwera et al., 2013). A full and detailed description of the analytical pipeline is provided in the Supplementary Text.

### DNA extraction and SNP genotyping

Genomic DNA for genotyping was extracted from lab bench grown 2 week old leaf tissue using Qiagen DNeasy maxi kits (Qiagen, Hilden, Germany). DNA quality was assessed using Nanodrop 2000 (Thermo Scientific, Massachusetts, USA) with a requirement of >1.8 for 260/280 and 260/230 ratios. DNA was quantified using Picogreen (Thermo Scientific, Massachusetts, USA). A total of 300 ng of lyophilised DNA per sample was sent to Geneseek (Neogen Corporation, UK) for Illumina HTS processing and HiScan chip imaging (Illumina, San Diego, USA). Genotyping at TraitGenetics was performed in house using the same technology and scanner type. SNP alleles were called using GenomeStudio Genotyping Module v2.0.2 (Illumina, San Diego, California). A total of 792 samples from various sources were genotyped, of which 148 overlapped with the 170 exome captured samples used for variant discovery (section SNP discovery). Agreement rates were then computed for these 148 samples between their GenomeStudio calls and the genotypes called by the variant calling pipeline in the exome capture data, using the approach detailed in section 1.7 of the Supplementary Data. In addition, DNA of 136 F_11_ RILs derived from a Golden Promise (GP) × Morex (Mo) cross (Liu et al., 2014), together with the two parental lines, were also processed as above. The resulting data were analysed as previously reported for 9k Illumina data (Comadran et al., 2012) through the use of Flapjack (Milne et al., 2010), JoinMap 4.0 (Van Ooijen, 2006) and Map Manager QTXb20 software (Manly et al., 2001). Initial analysis grouped the segregating SNPs in to linkage groups and then co-segregating SNPs excluded from the subsequent linkage mapping to increase computational efficiency. The resultant maps were then double checked and the co-segregating SNPs included back into the dataset in their calculated map position. A comparison with previous mapping in this population (Liu et al., 2014) allowed the checking of the marker coverage in the genetic interval on 5H that contains *HvDEP1*, the causal gene of *ari-e* (Wendt et al., 2016) that segregates in this population.

### SNP annotation

The 6,951 markers from the 9k chip were mapped onto the 2017 pseudomolecules genome assembly to supplement their existing genetic map positions with physical positions. Command line BLASTN version 2.6.0+ (Altschul et al., 1990; Camacho et al., 2009) was used, limiting the output to a maximum of three subject sequences and three high-scoring segment pairs (HSPs) per subject. The output was filtered to retain only hits with ≥95% identity. Custom Java code was then used to select the best hit from this filtered list. If a SNP manifest had multiple hits and one hit had a higher bit score than the remainder, that hit was chosen. If there were multiple hits and two or more hits from the top of the list had equal bit scores, the SNP was assigned to the “ambiguously mappable” category and all of its potential locations were exported. The code then checked the positions predicted by BLAST against the full list of all SNP positions in the exome capture, assuming that if both the predicted location and the type of polymorphism (e.g., A/G) matched, this would add additional confidence to SNP placement. Based on this, the SNPs were either annotated as “validated” or “unvalidated.”

All SNPs in the final design were annotated for functional effects using SnpEff 3.6b build 2014-05-01 (Cingolani et al., 2012), based on the gene model annotation provided with the barley pseudomolecules (Beier et al., 2017; Mascher et al., 2017).

## Results

### Germplasm for SNP discovery

Principal Coordinate Analysis, based on a simple matching pairwise matrix of 394 diverse domesticated barley accessions that had previously been characterized with 6,917 polymorphic SNPs from the 9k iSelect array, revealed 5 major groups: (i) spring 2-rowed types; (ii) spring 6-rowed types; (iii) winter 2-rowed types; (iv) winter 6-rowed types and (v) older European (mainly spring) types. From these groups, we used available background information, including pedigree, breeder and year of release, to select 148 representative accessions. We then added to these 148 lines another 22 old UK and Northern European landrace accessions without prior 9k iSelect SNP information, which made for a total of 170 lines (Table 1, Supplementary Table 1). These 170 accessions were used to identify potential SNPs for the 50k assay design (Figure 1).

**Table 1:** Composition of the 170 barley spring and winter germplasm lines chosen for the 50k SNP design.

**Breeding date**	**Springs**		**Winters**	
	**2-rowed**	**6-rowed**	**2-rowed**	**6-rowed**
1920–1940				1
1940–1960	4	1	0	1
1960–1980	14	2	1	2
1980–2000	66	3	18	6
2000–2010	24	5	12	3
Unknown	5	1		1
Total	113	12	31	14

### SNP selection for inclusion on the array

Barley whole genome EC data was made available for each of the170 lines for the purposes of SNP discovery and iSelect development. The complete EC dataset will be described elsewhere (Waugh et al., unpublished). After strict processing (see Supplementary Text) a total of 519,742 robust SNPs were identified as candidates for assay development. Manifest sequences (SNP plus 60 bp flanking sequence either side) for these 519,742 SNPs were then submitted to Illumina's Assay Design Tool (ADT, http://support.illumina.com/array/array_software/assay_design_tool.html) to prioritise candidates for inclusion on the array. In total, 295,642 SNPs passed the ADT criteria and a shortlist of SNPs to be included on the array was made on the basis of a trade-off between maximal sample representation and gene coverage. First, designable SNPs were filtered by including only those with ≤20% missing samples and a design score of ≥0.6, and removing duplicates based on association with genes (according to SnpEff annotation). This left 16,957 SNPs, representing single genes, ensuring maximum possible genic representation. Further SNPs were then added taking account of the relationship between the genetic and physical map in barley (Comadran et al., 2012; The International Barley Genome Sequencing Consortium, 2012). In barley, the pericentromeric regions are almost devoid of recombination (Mascher et al., 2017), and large numbers of markers cover a very small genetic interval. These are of limited use in genetic analyses as most will be in complete linkage disequilibrium and thus of low information value. This was compensated for by selectively adding more SNPs from the distal regions of chromosomes.

**Figure 2:**
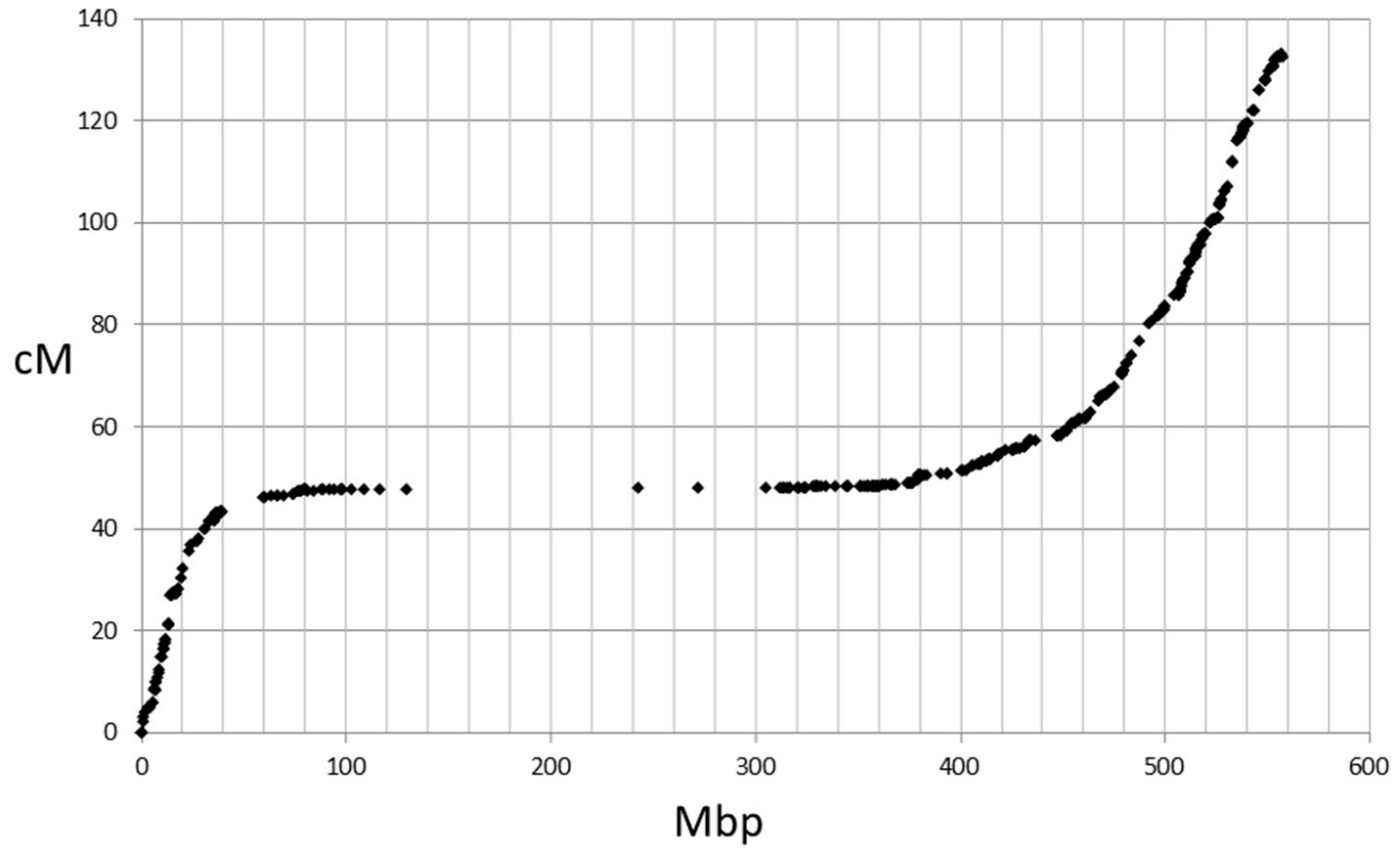
Plot of 9k SNP marker genetic map positions vs. physical map positions for chromosome 1H.

**Figure 3:**
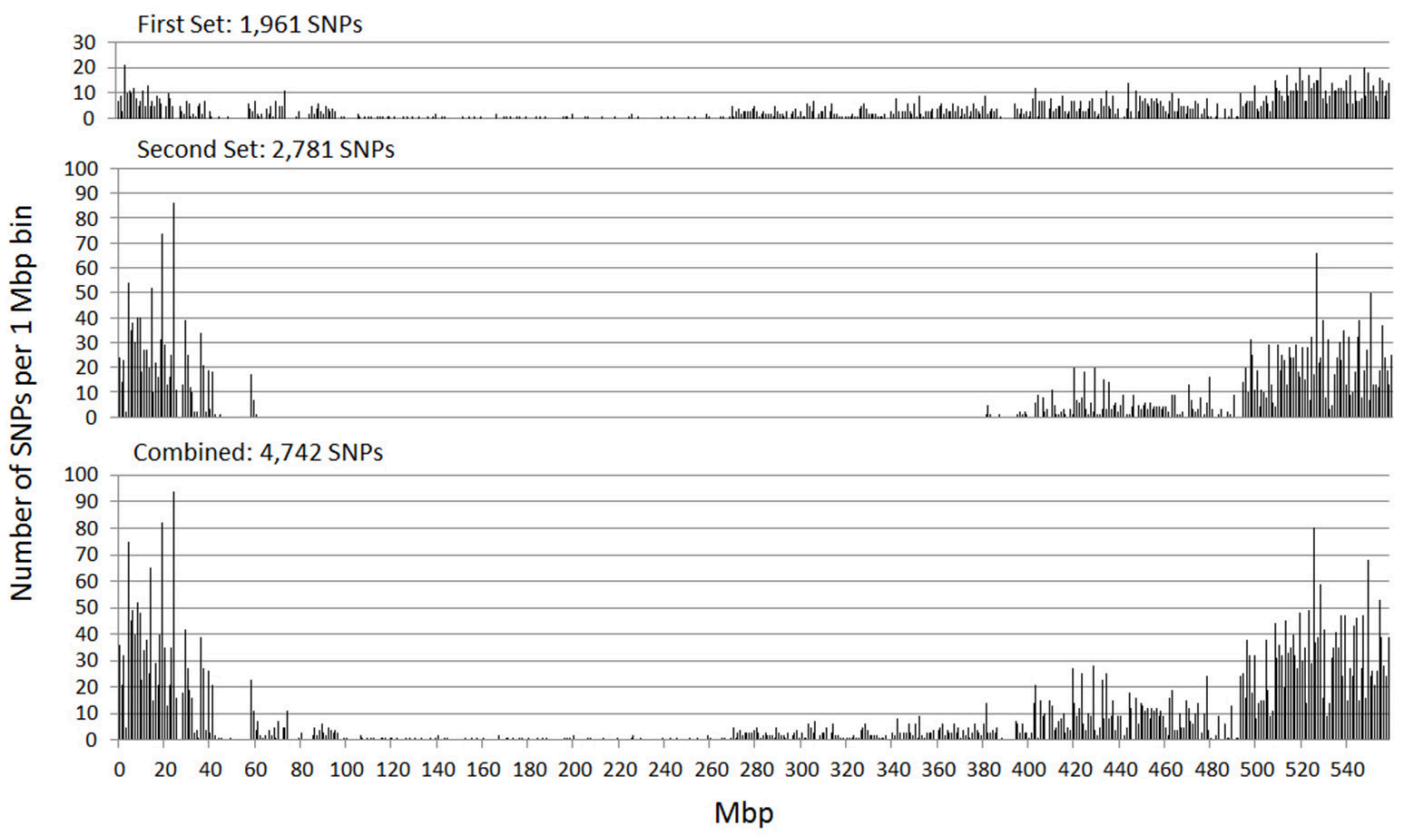
Physical distribution of the SNPs in the initial SNP set, the additional set targeted at markers in the distal chromosome regions, and the combination of these two sets (chromosome 1H).

The genetic map positions of the 9k SNPs were plotted on a Morex × Barke RIL population against the calculated physical positions of these SNPs (1H, Figure 2). These plots allowed each chromosome to be empirically partitioned into five sections relating to: (i) short arm with high recombination (e.g., 1H 0–30 Mb); (ii) short arm low recombination (1H 30–60 Mb); (iii) pericentromeric region (1H, 60–380 Mb); (iv) long arm low recombination (1H, 380–470 Mb) and; (v) long arm high recombination (1H, 470–560 Mb). SNPs were then selected from regions (i), (ii), (iv) and (v) in proportion to their representation on the genetic map (40, 7, 18, and 66 cM respectively in the case of 1H), intentionally skewing SNP choice to the distal chromosome arms. Similar constraints were used as before (score >0.8, missing samples <5%), but this time allowing 2 SNPs/gene. Constraints were relaxed slightly (score >0.6 and missing samples <20%) for the short arm of 5H because of the particularly high genetic::physical map ratio. This second set totalled 26,091 SNPs. The physical distribution of the SNPs in the two sets and the combined set for chromosome 1H is shown in Figure 3.

To provide backward compatibility with the existing 9k SNP chip, 6,951 functional markers were included that had proven reliable and informative across tens of thousands of genotyped samples (M. Ganal, unpublished data). These legacy markers were designed to keep the sequence orientation and allele calling identical to the 9k array to ensure direct comparability of the genotype data.

The shortlisted SNPs from the EC data (*n* = 43,048) were then screened for potential overlaps with those from the 9k set using standalone command line BLASTN (Altschul et al., 1990; Camacho et al., 2009), with SNP manifests of the 9k SNP set as queries, and manifests from the new set as subjects, with SNP polymorphisms encoded using IUPAC ambiguity codes. The BLAST results were filtered to leave hits with ≥98% query coverage and ≥95% identity. This identified 732 duplicates, which were removed, leaving 42,316 new SNPs. Combining these with the 6,951 9k SNPs produced a total of 49,267 SNPs in the final design, which was subsequently submitted to Illumina for chip fabrication. section Array performance below contains details of how the number of markers in the design translated into actual numbers of markers on the chip itself. Table 2 summarises the numbers of SNPs during the various stages of the selection process.

**Table 2:** Summary of SNP numbers during the selection process.

**SNP set description**	**# SNPs**
Total number called in 170 sample set	20,560,627
After filtering	528,439
Submitted to Illumina ADT (chr 1H-7H only)	519,742
Passed ADT analysis	295,642
Max. gene coverage set	16,957
Max. chromo coverage + gene evenness set	26,091
Total number from exome capture pre redundancy check	43,048
Overlap between 9k and EXCAP set	732
Legacy markers in design carried forward from 9k chip	6,951
Exome capture markers in design post redundancy check	42,316
Final number for array design	49,267
Total number of working assays on 50k chip	44,040 (6,251 from 9k + 37,789 from EXCAP)
Total number of scorable assays on 50k chip	43,461

### SNP annotation

To provide consistent annotation and physical map coordinates for all markers, the legacy markers from the 9k chip were mapped onto the 2017 pseudomolecules genome assembly, as they previously only had genetic map positions. Of the 6,251 working 9k markers on the 50k chip, 6,094 (97.5%) were mappable to a position on the pseudomolecules. Of these, 4,500 (73.8%) were validated by a corresponding SNP in the exome capture data. The physical positions for all SNPs on the chip are available in Supplementary Table 2, along with the SNP effect annotation. Table 3 shows statistics of the SNP annotation by functional class and region respectively. The ratio of transitions to transversions was 1.92. SNP effect annotation was attached to 40,972 of the 44,040 working markers on the chip (93.0%), and this represented 29,415 unique gene models from the 2017 pseudomolecules annotation (mean = 1.39 SNPs per gene).

**Table 3:** SNP effect annotation for the final set of SNPs on the 50k chip by type/region.

**SNP Type**	**Percent%**
Intron	28.56
Downstream	21.93
Upstream	13.80
UTR 3 prime	11.39
Synonymous coding	8.00
Non-synonymous coding	7.48
UTR 5 prime	4.88
Splice site region	1.95
Start gained	0.92
Intergenic	0.80
Stop gained	0.12
Stop lost	0.06
Splice site acceptor	0.05
Splice site donor	0.04
Synonymous stop	0.02
Start lost	0.01
Non-synonymous start	0.00

### Array performance

The final set of 49,267 SNPs in the array design converted into 44,040 working assays on the chip itself (= 89.4% conversion rate). Of these, 98.6% (43,461) were scorable in GenomeStudio, while 579 were eliminated due to insufficient cluster cut-off or other scoring problems. For the 6,951 SNP markers carried forward from the 9k array for design, 700 did not produce a working assay and were hence excluded, leaving 6,251 working legacy 9k markers on the 50k chip (Table 2). Across the 792 samples analysed, 97.3% of the 43,461 functional markers were polymorphic.

### Genotype call validation

**Figure 4:**
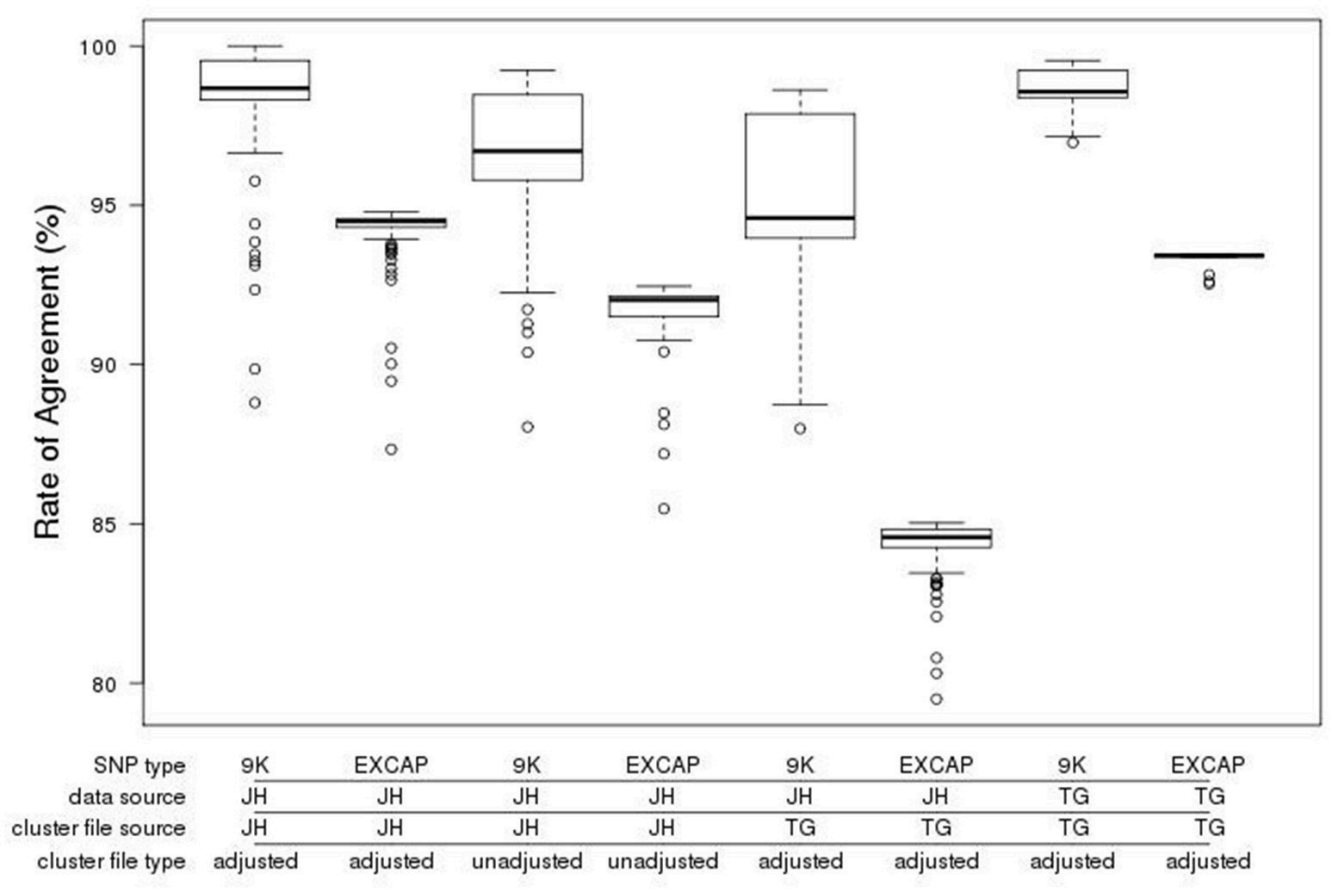
Boxplot of genotype call agreement rate (%) from the comparison of calls from the exome capture variant calling and the GenomeStudio calls for the accessions genotyped with the new 50k chip. The data shown is categorised by the provenance of the SNPs (9k = SNPs from the existing 9k chip, EXCAP SNPs = SNPs from exome capture data that are new and exclusive to the 50k chip), the source and type of cluster file (JH, James Hutton; TG, TraitGenetics, adjusted vs. unadjusted) and the source of the 50k genotype call set used (JH vs. TG). Bold horizontal lines represent the median, box boundaries upper and lower quartiles, whiskers maxima and minima, and open circles represent outliers.

To provide a measure of quality for the 50k array we genotyped 148 of the 170 barley accessions used for the variant discovery, for comparison with the existing genotype calls derived from the EC variant calls. The Illumina GenomeStudio software used for calling genotypes is designed to generate three clusters for each genotype (AA, BB, AB). However, barley is an inbreeding crop and the lack of heterozygous alleles can frequently lead to miscalls. Manual cluster curation by visual verification was therefore carried out, enabling optimum cluster coverage for each SNP according to the allelic distribution within GenomeStudio. Following adjustment of the cluster file, the genotyped individuals were rescored, generating mean agreement rates of 98.1 and 93.9% for 9k and the original EC SNPs respectively (Figure 4).

To assess technical variation across equipment and sites, manual cluster adjustment was also carried out at TraitGenetics (Gatersleben, Germany) on an independent set of 2,535 accessions, varieties and segregating material. We then scored the 148 samples genotyped at the James Hutton Institute, also with the manually adjusted TraitGenetics cluster file. This resulted in a decrease in mean agreement rates to 94.7 and 84.0% for 9k and EC respectively (Figure 4). A subset of the samples genotyped at TraitGenetics that overlapped with our exome captured samples (*n* = 16) scored with the manually adjusted TraitGenetics cluster file fared substantially better at 98.5 and 93.3% for 9k and EXCAP SNPs respectively. This indicates that there is some variation in the cluster distribution generated by individual Illumina scanners. The implication of this is that sharing adjusted cluster files is a good start but might require further adjustments to the local equipment.

### Genetic mapping

Processing 136 Golden Promise × Morex (GP × Mo) RILs, along with the two parental lines, resulted in data for 44,040 SNPs in the population. Of these, 29,413 SNPs were either monomorphic or had scoring issues (null or heterozygote calls in the parental lines). The segregation patterns of the remaining 14,627 SNPs were analysed, and co-segregating SNPs were excluded from the dataset to increase computational efficiency of subsequent linkage mapping, leaving 1,157 SNPs with unique segregation patterns. The genotypic data indicated that four of the RILs were duplicates and two others had excessive levels of heterozygosity, suggesting recent outcrossing. These six lines were excluded and the remaining 130 RILs used to develop a genetic linkage map using JoinMap 4.0 (Van Ooijen, 2006). This resulted in 14,626 SNPs being mapped to the seven barley chromosomes after re-incorporation of the co-segregating SNPs (Table 4). The genetic map totalled 914.0 cM, with all distal markers being within 2 Mbp of the end of the respective chromosome, with the exception of 3HL (3.48 Mbp). Although the resultant genetic map was high density, there remained 11 intervals of greater than 5 cM, relating to regions of higher recombination rate or identity by state.

**Table 4:** Summary of the genetic map derived from the GP × Mo RIL population.

**Chromosome**	**Length (cM)**	**No. SNPs**
1H	113.7	1,730
2H	146.1	2,927
3H	127.3	2,160
4H	118.4	1,371
5H	179.3	2,299
6H	116.0	1,991
7H	110.2	2,148
Total	914.0	14,626

**Figure 5:**
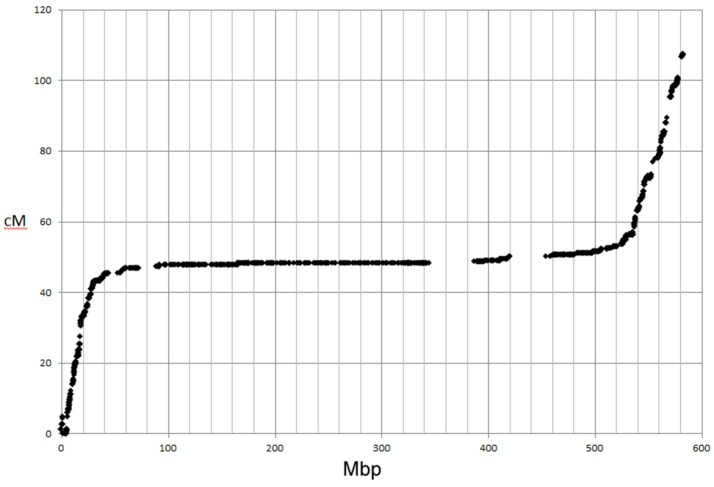
Comparison of genetic map and physical positions of SNPs on chromosome 6H mapped in GPxMo RIL population.

The ordering of the SNPs in the linkage map was almost completely in accordance with their expected physical order. As expected for large cereal genomes, the relationship between the genetic and physical distance was not linear. Figure 5 shows the physical:genetic relationship for 6H, with most recombination in the distal ends of the short and long arms. There were instances where neighbouring SNPs were transposed in the linkage map relative to their expected physical order, when a recombination event separated SNPs derived from different contigs within a BAC (Supplementary Table 3). The genetic map also showed two larger scale inconsistencies with the physical sequences at the distal ends of 4HL and 6HS, which could indicate some minor issues with the physical assembly (Supplementary Table 3). This was supported by a comparison of the genetic map with previous SNP mapping in other bi-parental populations (Close et al., 2009; Comadran et al., 2012; Supplementary Table 3). These comparisons also supported the map positions found for 51 of the 9k SNPs, for which there was no evident physical position. A small number of SNPs (44/14,626; 0.3%) mapped to unlinked positions compared to their expected physical location (Supplementary Table 3).

**Figure 6:**
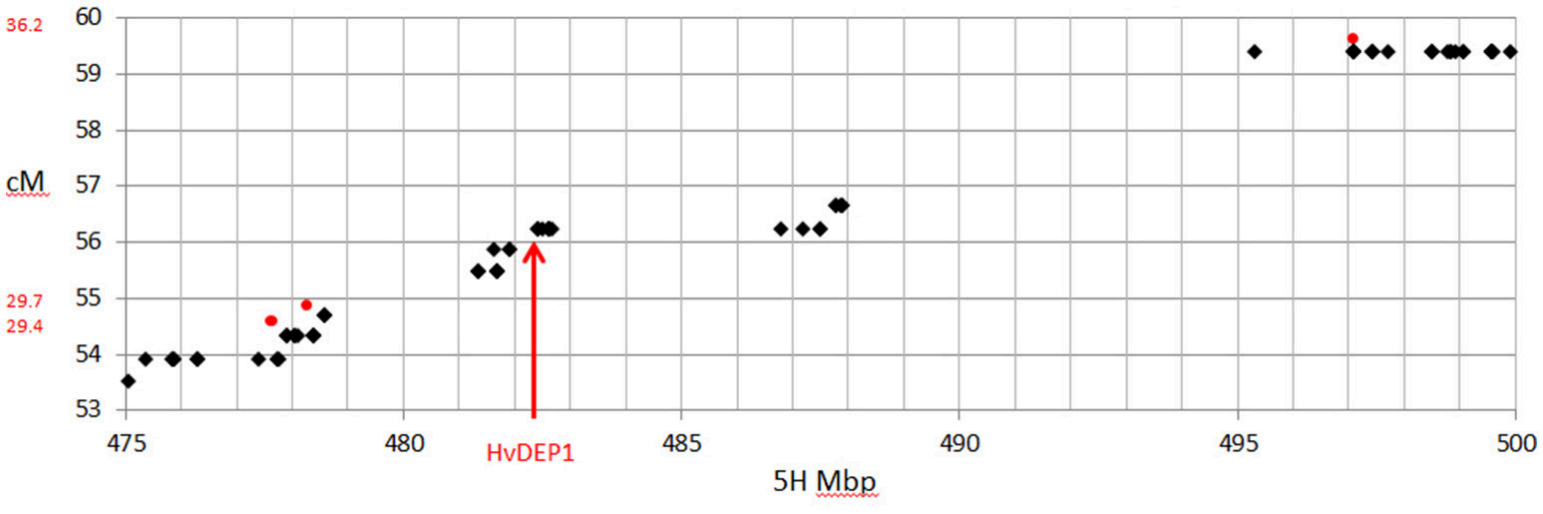
Comparison of genetic map and physical position on chromosome 5H around the position of *HvDEP1*, the causal gene underlying the ari-e.GP mutant phenotype mapped in the GP × Mo RIL population. SNPs from the 50k are shown as black diamonds and the three GBS SNPs are shown as red circles.

Comparison of the maps derived here and one previously generated from GBS data (Liu et al., 2014) showed a slight decrease in the total genetic length (935.5 cM compared to 914.0 cM), with a considerably higher number of mapped SNPs (1,596 from GBS compared to 14,626 from this array). The increased density of high quality markers allowed better coverage of the genome and filled in some gaps in the published GBS map for this population. This included a 7.2 cM gap on 5HL, that previously precluded fine mapping of the *ari-e.GP* locus (Liu et al., 2014). Using the new platform, the gap is populated with 43 SNPs that define 9 recombination events, including two that flank *HvDEP1*, the causal gene of *ari-e* (Wendt et al., 2016), narrowing it to a 0.8 cM interval including 8 genes (Figure 6).

### Online database with marker information

An online resource was developed to store background data such as the SNP source sequence, locus name, sequence orientation, SNP effects data and any additional information known on each locus. This resource is based on the Germinate data infrastructure (Shaw et al., 2017) and allows users to search and retrieve background design information for each SNP (49,267 in total) on the barley 50k SNP array. Users can search for individual marker names to retrieve additional information or download the entire dataset for browsing in text or Excel. Information is included for the 9k SNP array (Comadran et al., 2012) within this database. This resource is freely available from https://ics.hutton.ac.uk/50k.

## Discussion

The performance statistics of the new barley 50k iSelect platform were comparable to the previous 9k chip in terms of failed assays (10.6 vs. 10.9% respectively) and the percentage of polymorphic markers (97.3 vs. 94.7% respectively), although the latter figures need to be interpreted with caution as they are not based on the same set of lines in each case, and polymorphism rates will obviously vary with the types of samples assessed. The performance statistics are comparable to those reported in other recent crop genotyping platforms (Sim et al., 2012; Bianco et al., 2014; Chen et al., 2014). The high validation rate for the 50k genotype calls is encouraging and shows that SNP data derived from a variety of sources and platforms is highly accurate. Some variation was observed between the subsets of SNPs. The legacy markers carried forward from the existing 9k chip performed better in the genotype call comparison (Figure 4) and this is likely due to the selection procedure for the legacy markers, which singles out assays that have performed well previously. This is supported by the fact that the original BOPA markers, that had been through this selection process twice, performed even better than the remainder derived from the 9k array.

The validation procedure highlights issues around the scoring of genotypes in Illumina's GenomeStudio software. Datasets generated at two genotyping facilities, along with the associated adjusted cluster files from the two sites, were compared. The results showed that manually adjusted cluster files perform significantly better than their unadjusted equivalents, but it was also apparent that using an adjusted cluster file to analyse raw data generated at different genotyping facilities leads to a deterioration, rather than improvement, of results. This is due to slight differences in the hardware used to generate raw call data resulting in somewhat different signal intensities, and implies that each genotyping facility should carry out their own adjustments to a cluster file generated in a different facility, rather than using it without further curation.

The computational approach taken here for the discovery of the novel set of SNPs (see Supplementary Text) utilized the highly sophisticated Genome Analysis Toolkit (GATK) and its recommended Best Practices pipeline. This approach is technically involved and computationally costly, in terms of CPU hours, memory consumption and storage of the resulting GVCF files, which are proprietary to GATK and consume roughly as much disk space again as the corresponding BAM files. However, the computational effort does appear to be vindicated by the accuracy of the variant calls, as benchmarking data from review articles suggests (Liu et al., 2013; Huang et al., 2015).

The germplasm used for generation of the new SNPs on the 50k chip was chosen carefully to represent a wide range of UK and other European elite cultivars drawn from a number of different collections and projects. This includes spring and winter cultivars, as well as two-rowed and six-rowed barleys. A total of 170 different lines was used, which captures a large amount of the total genetic variation that exists among modern European germplasm, maximizing the utility of the chip for barley geneticists and breeders, thus limiting the ascertainment bias associated with previous designs (Moragues et al., 2010). In addition, all the usable markers from the previous 9k chip were included, which will further enhance the utility of the chip for breeding purposes by providing backward compatibility when new germplasm is compared to legacy material.

To generate the new markers, the recently released barley pseudomolecules genome assembly (Beier et al., 2017; Mascher et al., 2017) was used. This has provided a reference sequence of unprecedented resolution and quality that has a substantially more intact gene space (Beier et al., 2017) than the previous assembly (The International Barley Genome Sequencing Consortium, 2012). In combination with exome capture technology, this allowed a set of high quality SNPs to be generated that are almost exclusively associated with genic regions. In addition, the gene model annotation of the new genome assembly enables both SNP effect predictions and functional annotation for the genes associated with the variants. This further increases the utility of the array for breeding purposes.

A targeted stratification approach was used to ensure that the new variants on the array provided both even coverage across genes (by limiting the numbers of SNPs per gene) and meaningful coverage across chromosomes. The pericentromeric regions of barley chromosomes are effectively devoid of recombination and hence any markers located in these regions are likely to be in linkage disequilibrium and consequently of little use to breeders. A two-step approach was used that first selected a single SNP per available gene, and then added to this a second set of SNPs that were preferentially drawn from the recombination-rich distal parts of chromosomes. This provides a SNP set that is both broad and even in terms of gene coverage, but also focused on SNPs that are as informative as possible.

Use of the 50k chip on the bi-parental GP × Mo RIL population (Liu et al., 2014) demonstrated its utility with the construction of a robust genetic map with 14,626 SNPs. The genetic map (914 cM) was slightly shorter than both standard and consensus SNP maps derived from other populations (Close et al., 2009; Comadran et al., 2012; The International Barley Genome Sequencing Consortium, 2012), but does accord with previous mapping in this population (Liu et al., 2014).

The completeness and quality of the segregation data facilitated construction of a map with the majority of individual recombination events delineated by flanking SNP markers. Given the coverage of the genome of the chip, some of the 11 gaps >5 cM could be ascribed to regions of monomorphism due to identity-by-state, despite the genetic distance between the UK two-rowed and US six-rowed parental lines, or to regions of high recombination rate, in particular on the short arm of 5H flanking the presumed NOR region (Dubcovsky and Dvorák, 1995).

Importantly, the SNP coverage of the genome represented on the 50k chip improved the coverage of the genetic map significantly compared to previous GBS work (Liu et al., 2014). In particular, the previous 7.2 cM gap on 5HL that precluded fine mapping of the *ari-e.GP* locus was well populated, with 43 new SNPs. This indicates that this is not a region of monomorphism as previously postulated (Liu et al., 2014), and that recombination events in this population could have been used to delineate the causal gene *HvDEP1* (Wendt et al., 2016) should such markers have previously been available. Similarly there are 110 additional SNPs mapped with the 50k chip between the BOPA1 SNPs, 11_20265 and 11_20392 that flank the ari-e.GP locus on the Derkado × B83-12/21/5 DH map (Wendt et al., 2016).

The genetic map derived from the GP × Mo RIL population validated the strategy developed here and the quality of the information used to construct the 50k chip, as well as the quality of the barley physical map (Mascher et al., 2017). Interestingly, the completeness and quality of the segregation data highlighted a number of instances where recombination between SNPs from neighbouring genes indicated that the linear order was not as expected. These were generally explicable in terms of the known relatively poorly defined fine-scale ordering of contigs within BACs. Some larger scale discrepancies between the genetic map ordering and pseudomolecules (distal regions in 4HL and 6HS) were supported by previous genetic mapping with common SNPs (Close et al., 2009; Comadran et al., 2012) and highlighted the utility and robustness of the 50k genotyping platform.

## Author contributions

MB carried out the variant calling and downstream analysis of the exome capture data. PR carried out the benchmarking of the GATK components. MG and JP selected 9k markers for inclusion on the 50k chip and generated data for the chip validation. JR selected lines for inclusion in the data analysis and with PH and LR generated the strategy for inclusion of markers on the chip. MM genotyped the set of lines used for validation and generated the adjusted cluster file for GenomeStudio. LR carried out the stratification of markers across chromosomes, the comparison of physical vs. genetic positions of the 9k legacy SNPs, and the genetic mapping in the GPxMo RIL population. PS generated the SNP marker database and web interface. WT curated the set of lines used for validation. RW coordinated and supervised the work. All authors contributed to drafting the manuscript and/or helped revise the manuscript.

### Conflict of interest statement

MG and JP are affiliated with TraitGenetics GmbH, a commercial provider of genotyping services offering the platform described here. This does not alter the authors' adherence to all editorial policies on sharing data and materials. No conflicting interests are declared by any of the other authors.

## Supplementary material

Supplementary Table 1 List of the 170 barley lines used for variant discovery.

Supplementary Table 2 List of physical positions for the SNP markers on the 50k chip.

Supplementary Table 3 Genetic map of the Golden Promise × Morex cross population genotyped with the new 50k chip.
