# Supplementary material for: Development and Evaluation of a Barley 50k iSelect SNP Array
Source: Front Plant Sci. 2017 Oct 17;8:1792. doi: 10.3389/fpls.2017.01792 (PMC5651081; doi:10.3389/fpls.2017.01792)
Supplement: Supplementary file 4 [file DataSheet1.DOCX]

Supplementary Material

Development and evaluation of a barley 50k iSelect SNP array

Micha Bayer^1*^, Paulo Rapazote-Flores^1^, Martin Ganal^2^, Pete Hedley^1^, Malcolm Macaulay^1^, Joerg Plieske^2^, Luke Ramsay^1^, Joanne Russell^1^, Paul Shaw^1^, William (Bill) Thomas^1^, Robbie Waugh^1^

^1^ The James Hutton Institute, Invergowrie, Dundee DD2 5DA, Scotland, UK

^2^ TraitGenetics GmbH, Am Schwabeplan 1b, Stadt Seeland OT Gatersleben, D-06466, Germany

*** Correspondence:**Micha Bayer
micha.bayer@hutton.ac.uk

# Detailed pipeline for SNP discovery and filtering

## General approach

The Illumina Exome Capture read processing was carried out in line with the recommendations made in the Genome Analysis Toolkit (GATK) Best Practices documentation (Van der Auwera et al., 2013). A shell script written in BASH (<https://www.gnu.org/software/bash/>) was used to implement the GATK Best Practices pipeline.

## Read mapping

The 2017 barley genome assembly was used as the reference sequence (Beier et al., 2017;Mascher et al., 2017), which represents each of the seven chromosomes as pseudomolecules up to ~770 Mbp in length. For each sample, the raw, untrimmed reads were mapped to the reference sequence separately using BWA-MEM v. 0.7.10 (<http://bio-bwa.sourceforge.net/bwa.shtml>). Using raw data is in line with the recommendations of the GATK Best Practices, which stipulates that reads are mapped untrimmed to enable accurate removal of read duplicates, which is based on matching start and end coordinates of untrimmed reads. Parts of reads that are mismatched with the reference sequence were soft-clipped in BWA-MEM and excluded from the variant analysis.

The BAM output was filtered with the bamtools toolkit (https://github.com/pezmaster31/bamtools) to remove reads that contained more than 4% mismatches, based on their alignment score (AS) flag in the SAM/BAM output. Mismatch cut-offs in read mapping are essential for the accuracy of downstream analysis as read mismapping caused by overly relaxed mismatch parameters can lead to dramatically increased false positive rates in variant calling (Ribeiro et al., 2015).

## BAM file preprocessing

The GATK toolkit Best Practices documentation (Van der Auwera et al., 2013) includes a number of BAM file preprocessing steps before the actual variant calling itself can take place. The first of these is the removal or marking of duplicate read pairs. To reduce storage requirements we opted for removal with samtools rmdup (Li et al., 2009). The second BAM file preprocessing step consists of a local realignment of reads around indels. This adjusts the placement of reads that have been aligned suboptimally around indels, and thereby removes base mismatches that could be misinterpreted as variants by the downstream variant caller. Both of the steps above are designed to keep the false positive SNP rate to a minimum (DePristo et al., 2011).

## Variant and genotype calling

The deduplicated, realigned BAM files were then run through the variant caller component of GATK, HaplotypeCaller, to produce an initial VCF output file. This was filtered using the vcffilter tool (<https://github.com/vcflib/vcflib#vcffilter>) to produce a second VCF file containing high quality variants with a variant quality score of >=20. This filtered VCF file was used as a truth set for the subsequent recalibration of base quality scores. In the absence of a publicly available benchmark dataset, calibration datasets are generated by means of a bootstrapping approach that produces a high quality call set. We found that a single iteration provided an acceptable balance between improved convergence and computation time.

Base quality score recalibration (BQSR) was then used to remove the positional and contextual bias found in raw base quality scores (DePristo et al., 2011). The recalibrated BAM files were then used as input for the second run of the HaplotypeCaller component, the output of which is in GVCF format (see <https://software.broadinstitute.org/gatk/guide/article?id=4017>). The recalibrated BAM files were visually spot-checked using the Tablet assembly viewer (Milne et al., 2010a;Milne et al., 2013).

## Batch generation and joint genotyper run

The GVCF files from all samples have to be combined into cohort files using the GATK’s CombineGVCFs component before being run through the joint genotyper (GenotypeGVCFs). For large numbers of samples, either step can produce a computational bottleneck in terms of CPU time and memory usage, and we therefore decided to first experimentally test different cohort sizes to establish the optimal batching strategy. One hundred randomly chosen samples from the exome capture dataset described in the main text were combined into cohorts of different sizes (range 5 – 50) in five different replicate runs to evaluate the compute resources required. Supplementary Figure 1 shows the results of this. A cohort size of 20 samples offered a good compromise between CPU time and memory consumption for both the cohort formation step and the genotype calling. Based on this, the 205 initial samples were batched into 11 cohorts and these were input into the joint genotyper to produce a VCF file.

## Variant filtering

The raw VCF output from the joint genotyper was subjected to stringent filtering to ensure that only true positive SNPs with good sample representation were selected for the final set to be used on the chip. The initial filtering was carried out with custom written Java code and the conditions required for a SNP to pass were as follows:

1. >= 8x coverage for >= 50% of the samples (to ensure robust SNP and genotype calls)
2. >= 95% of samples represented at SNP locus (for maximum sample representation)
3. >= 5% minor allele frequency at the level of the sample, i.e. counting sample genotypes rather than individual reads (to exclude SNPs based on very rare alleles)
4. >= 30 SNP quality score, equating to a >= 1/1000 chance of the SNP having been called in error (for SNP robustness)
5. >= 98% of samples homozygous (to reduce false positives by removing variants that are the result of read mismapping or Illumina systematic sequencing error (Meacham et al., 2011;Nakamura et al., 2011))
6. no indels

## Genotype visualization and quality control of lines

The filtered VCF file was converted into .map and .dat files suitable for import into the Flapjack genotype visualization software (Milne et al., 2010b). Flapjack can be used to highlight heterozygotes in the genotype calls and can thus provide a broad overview of the overall consistency of the data. Cultivated barley is a diploid inbred species and therefore expected to be largely homozygous, with only small pockets of heterozygosity remaining that are mostly cultivar-specific. The pattern of heterozygosity observed in the visualization (Supplementary Figure 2) reflects this as short tracts of light horizontal lines, sparse in occurrence and randomly distributed across regions and samples. Any accumulation of heterozygosity within lines (=rows) indicates potential sample contamination, whereas any marker (=column) with a large proportion of heterozygous genotype calls indicates a potential false positive SNP caused by mismapping or systematic sequencing error (Meacham et al., 2011;Nakamura et al., 2011). To quantitate rates of heterozygosity in individual lines (across markers), we used a BASH shell script to count heterozygous genotype calls in each line.

We also validated the identity of lines in the exome capture using an existing set of genotype calls from the barley 9k iSelect platform (Comadran et al., 2012). Many of the exome captured elite cultivars described in section 2.1 had been genotyped with the BOPA1 SNP platform (Close et al., 2009) and then again with the iSelect SNP platform, and thus the common set of SNPs across both platforms was used to check the authenticity of both sets of genotyping data. Where significant differences existed between the two, the sources of seed were checked to establish if they were due to naming problems where breeders re-use old names of varieties that are no longer commercialised and then obvious morphological differences such as row type, growth habit, anthocyanin pigmentation, and rachilla, and glaucosity used to establish which version was which and thus the true elite cultivar. In cases where varieties could not be distinguished in this way, the position of the line within a dendrogram formed by hierarchical clustering analysis relative to both its pedigree and growth habit was used to establish which version was the correct elite line. Where we had the ‘wrong’ version of a line, we re-sourced seed from a separate collection, if possible, and re-genotyped the line. This provided a set of 1159 curated genotypes for 1159 lines.

We then compared the genotype calls from the curated set described above with the genotype calls from the exome capture data. First, the 9k iSelect manifest sequences were mapped to the pseudomolecules reference sequence using BWA-MEM. Java code was developed to extract the positions of the 9k chip SNPs that were mapped, and to compare the benchmark genotypes to the genotypes stored in the filtered VCF file resulting from the exome capture variant calling. For each benchmark SNP, the code recorded a match or a mismatch, resulting in a final figure of % agreement across all markers, for each line assayed. The assumption with this type of comparison is that agreement between these two independently generated datasets indicates that a given SNP is real and its assay is performing as expected.

## Sample QC and final sample set

Given that low genotype call agreement rates and high heterozygosity are indicators of sample mixup and/or cross-contamination, we plotted these variables against each other to identify samples that should be excluded from further analysis (Supplementary Figure 3). This produced a relatively coherent cluster of samples in the top left of the plot which represents the higher end of the % identity range and the lower end of the heterozygosity range (group 1). Another cluster was discernible below this that contained samples of low % identity but also low heterozygosity (group 2), leaving a third group of samples to the right with both low % identity and high % heterozygosity values (group 3). The latter two groups of samples were deemed unsuitable for further analysis as they presumably had either been cross-contaminated or swapped with other samples, and accordingly we set cut-offs of >95% for identity and <5% for heterozygosity for a sample to be retained. Based on this analysis, we removed the cross-contaminated and/or swapped samples from the analysis (n = 35) and repeated the GATK joint genotyper run with the remaining 170 good samples (the remainder of the analysis described here pertains to this reduced set of samples).

This resulted in 20,560,627 variants in the raw VCF output. To generate a sufficiently large pool of SNPs to choose from for the chip design, the filtering parameters on this run were relaxed to allow for more missing samples. Instead of requiring >= 8x coverage for >= 50% of the samples, we opted for >= 4x coverage for >= 30% of the samples, and allowed 70% missing samples. This left 528,439 SNPs post-filtering which were carried forward for further analysis.

We extracted SNP quality scores (QUAL) and total read coverage from the filtered VCF file. The phred-based (Ewing and Green, 1998) SNP quality score (<https://vcftools.github.io/specs.html>) ranged from 30 to 1,392,214 (mean 25,106), equating to probabilities of 1E-3 and 1E-139221 (mean 1E-2511), respectively, of the SNP having been called in error. This illustrates the high degree of robustness conferred by the strict filtering applied here. The total read coverage at SNP positions ranged from 134 to 38,970 (mean 1,909).

## SNP scoring with the Assay Design Tool

SNP manifests for the filtered SNPs were extracted from the reference sequence using custom Java code. This extracted 60 base pairs (bp) of the reference sequence either side of each SNP location. SNPs on ChrUn were not taken forward for further analysis. A total of 519,742 SNP manifests were then submitted to Illumina’s Assay Design Tool for scoring (ADT, see http://support.illumina.com/array/array_software/assay_design_tool.html).The scoring process takes into account any potential source of failure for a SNP assay, such as neighbouring variants in the flanking regions, and fails a SNP if such features are detected. The remainder of SNPs is given a design score between 0 and 1 (1 = best). This score reflects the likelihood of success for a given assay. Of the 519,742 manifests submitted for scoring, 295,642 passed the ADT screening for low sequence complexity and degenerate nucleotides in the assay design region, and were thus potential candidates for inclusion on the chip. Of the remainder, the vast majority failed due to variants in the flanking regions (n = 222,809), and a small number failed due to being duplicated (n = 1,289) or because the top/bottom strand could not be determined (n = 2). The design score distribution of the SNPs that passed filtering is shown in Supplementary Figure 4.


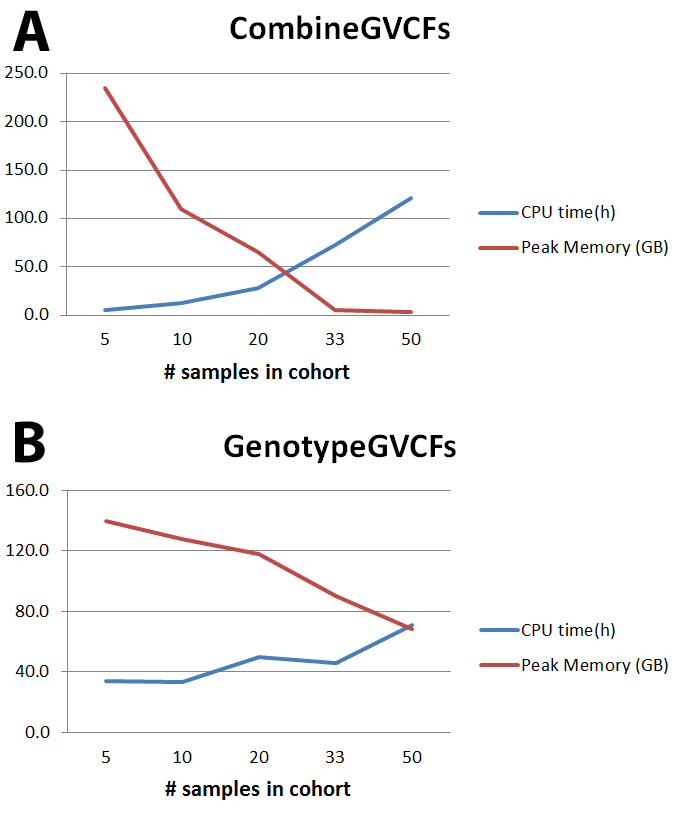


**Supplementary Figure 1.** CPU time and peak memory requirements as a function of the number of samples combined into cohorts when invoking the GATK’s CombineGVCFs and GenotypeGVCFs components.


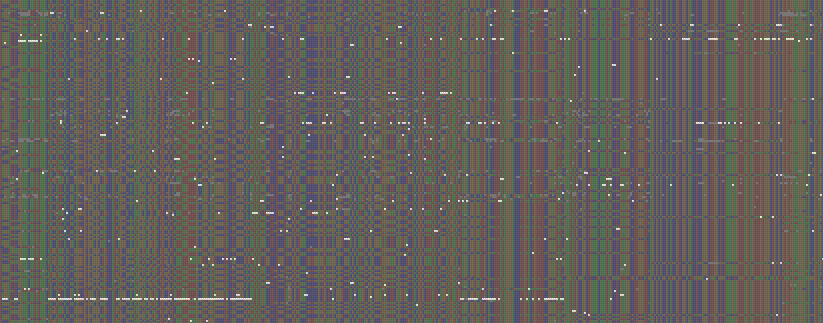


**Supplementary Figure 2.** Flapjack screenshot showing visual representation of genotypes in exome capture based variants. Highlighted data points indicate heterozygous genotype calls. Lines are arranged in rows, markers in columns. Several lines can be seen that show a higher than normal proportion of heterozygous calls, indicating a potential problem with sample contamination.


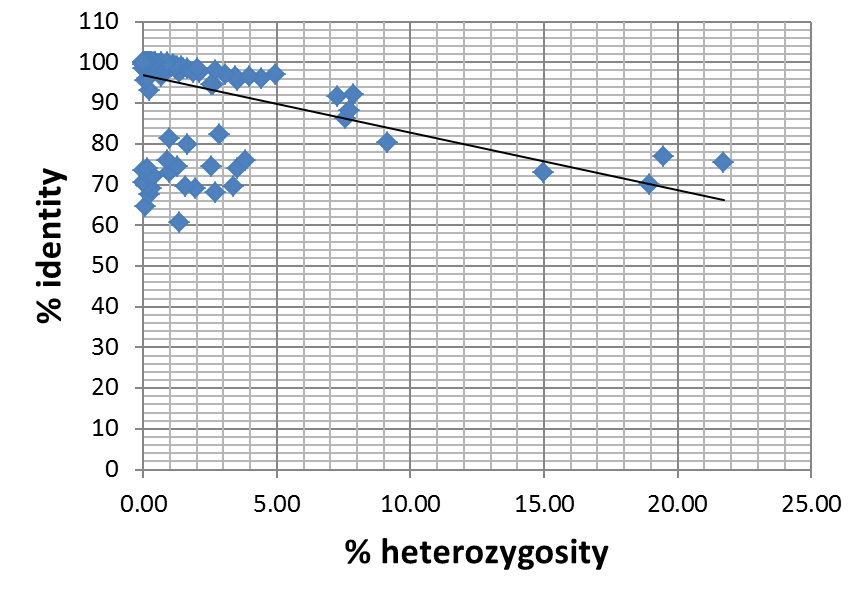


**Supplementary Figure 3.** Scatter plot of % genotype call agreement rate (“% identity”) versus % heterozygosity of the 205 barley lines used for variant calling, based on the filtered VCF data as described above. The % identity data were obtained by comparing the VCF genotypes to a curated set of 9k iSelect genotype calls which had been corroborated by phenotypic and other evidence over a number of years. The % heterozygosity data reflect, for each line, the percentage of heterozygous genotype calls across all markers in its exome capture data.


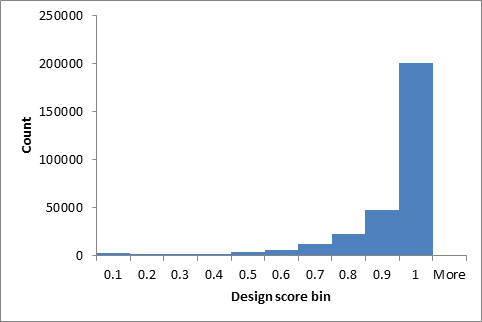


**Supplementary Figure 4.** Histogram of the design scores of SNPs that passed filtering.

# References

Beier, S., Himmelbach, A., Colmsee, C., Zhang, X.-Q., Barrero, R.A., Zhang, Q., Li, L., Bayer, M., Bolser, D., Taudien, S., Groth, M., Felder, M., Hastie, A., Šimková, H., Staňková, H., Vrána, J., Chan, S., Muñoz-Amatriaín, M., Ounit, R., Wanamaker, S., Schmutzer, T., Aliyeva-Schnorr, L., Grasso, S., Tanskanen, J., Sampath, D., Heavens, D., Cao, S., Chapman, B., Dai, F., Han, Y., Li, H., Li, X., Lin, C., Mccooke, J.K., Tan, C., Wang, S., Yin, S., Zhou, G., Poland, J.A., Bellgard, M.I., Houben, A., Doležel, J., Ayling, S., Lonardi, S., Langridge, P., Muehlbauer, G.J., Kersey, P., Clark, M.D., Caccamo, M., Schulman, A.H., Platzer, M., Close, T.J., Hansson, M., Zhang, G., Braumann, I., Li, C., Waugh, R., Scholz, U., Stein, N., and Mascher, M. (2017). Construction of a map-based reference genome sequence for barley, Hordeum vulgare L. *Scientific Data* 4**,** 170044. doi: 10.1038/sdata.2017.44

Close, T.J., Bhat, P.R., Lonardi, S., Wu, Y., Rostoks, N., Ramsay, L., Druka, A., Stein, N., Svensson, J.T., Wanamaker, S., Bozdag, S., Roose, M.L., Moscou, M.J., Chao, S., Varshney, R.K., Szűcs, P., Sato, K., Hayes, P.M., Matthews, D.E., Kleinhofs, A., Muehlbauer, G.J., Deyoung, J., Marshall, D.F., Madishetty, K., Fenton, R.D., Condamine, P., Graner, A., and Waugh, R. (2009). Development and implementation of high-throughput SNP genotyping in barley. *BMC Genomics* 10**,** 582. doi: 10.1186/1471-2164-10-582

Comadran, J., Kilian, B., Russell, J., Ramsay, L., Stein, N., Ganal, M., Shaw, P., Bayer, M., Thomas, W., Marshall, D., Hedley, P., Tondelli, A., Pecchioni, N., Francia, E., Korzun, V., Walther, A., and Waugh, R. (2012). Natural variation in a homolog of Antirrhinum CENTRORADIALIS contributed to spring growth habit and environmental adaptation in cultivated barley. *Nature Genetics* 44**,** 1388-1392. doi: 10.1038/ng.2447

Depristo, M.A., Banks, E., Poplin, R., Garimella, K.V., Maguire, J.R., Hartl, C., Philippakis, A.A., Del Angel, G., Rivas, M.A., Hanna, M., Mckenna, A., Fennell, T.J., Kernytsky, A.M., Sivachenko, A.Y., Cibulskis, K., Gabriel, S.B., Altshuler, D., and Daly, M.J. (2011). A framework for variation discovery and genotyping using next-generation DNA sequencing data. *Nature Genetics* 43**,** 491-+. doi: 10.1038/ng.806

Ewing, B., and Green, P. (1998). Base-Calling of Automated Sequencer Traces Using Phred. II. Error Probabilities. *Genome Research* 8**,** 186-194. doi: 10.1101/gr.8.3.186

Li, H., Handsaker, B., Wysoker, A., Fennell, T., Ruan, J., Homer, N., Marth, G., Abecasis, G., Durbin, R., and Genome Project Data Processing, S. (2009). The Sequence Alignment/Map format and SAMtools. *Bioinformatics* 25**,** 2078-2079. doi: 10.1093/bioinformatics/btp352

Mascher, M., Gundlach, H., Himmelbach, A., Beier, S., Twardziok, S.O., Wicker, T., Radchuk, V., Dockter, C., Hedley, P.E., Russell, J., Bayer, M., Ramsay, L., Liu, H., Haberer, G., Zhang, X.-Q., Zhang, Q., Barrero, R.A., Li, L., Taudien, S., Groth, M., Felder, M., Hastie, A., Šimková, H., Staňková, H., Vrána, J., Chan, S., Muñoz-Amatriaín, M., Ounit, R., Wanamaker, S., Bolser, D., Colmsee, C., Schmutzer, T., Aliyeva-Schnorr, L., Grasso, S., Tanskanen, J., Chailyan, A., Sampath, D., Heavens, D., Clissold, L., Cao, S., Chapman, B., Dai, F., Han, Y., Li, H., Li, X., Lin, C., Mccooke, J.K., Tan, C., Wang, P., Wang, S., Yin, S., Zhou, G., Poland, J.A., Bellgard, M.I., Borisjuk, L., Houben, A., Doležel, J., Ayling, S., Lonardi, S., Kersey, P., Langridge, P., Muehlbauer, G.J., Clark, M.D., Caccamo, M., Schulman, A.H., Mayer, K.F.X., Platzer, M., Close, T.J., Scholz, U., Hansson, M., Zhang, G., Braumann, I., Spannagl, M., Li, C., Waugh, R., and Stein, N. (2017). A chromosome conformation capture ordered sequence of the barley genome. *Nature* 544**,** 427-433. doi: 10.1038/nature22043

<http://www.nature.com/nature/journal/v544/n7651/abs/nature22043.html#supplementary-information>

Meacham, F., Boffelli, D., Dhahbi, J., Martin, D.I.K., Singer, M., and Pachter, L. (2011). Identification and correction of systematic error in high-throughput sequence data. *Bmc Bioinformatics* 12. doi: 10.1186/1471-2105-12-451

Milne, I., Bayer, M., Cardle, L., Shaw, P., Stephen, G., Wright, F., and Marshall, D. (2010a). Tablet--next generation sequence assembly visualization. *Bioinformatics* 26**,** 401-402. doi: 10.1093/bioinformatics/btp666

Milne, I., Shaw, P., Stephen, G., Bayer, M., Cardle, L., Thomas, W.T., Flavell, A.J., and Marshall, D. (2010b). Flapjack--graphical genotype visualization. *Bioinformatics* 26**,** 3133-3134. doi: 10.1093/bioinformatics/btq580

Milne, I., Stephen, G., Bayer, M., Cock, P.J.A., Pritchard, L., Cardle, L., Shaw, P.D., and Marshall, D. (2013). Using Tablet for visual exploration of second-generation sequencing data. *Briefings in Bioinformatics* 14**,** 193-202. doi: 10.1093/bib/bbs012

Nakamura, K., Oshima, T., Morimoto, T., Ikeda, S., Yoshikawa, H., Shiwa, Y., Ishikawa, S., Linak, M.C., Hirai, A., Takahashi, H., Altaf-Ul-Amin, M., Ogasawara, N., and Kanaya, S. (2011). Sequence-specific error profile of Illumina sequencers. *Nucleic Acids Research* 39**,** e90. doi: 10.1093/nar/gkr344

Ribeiro, A., Golicz, A., Hackett, C., Milne, I., Stephen, G., Marshall, D., Flavell, A., and Bayer, M. (2015). An investigation of causes of false positive single nucleotide polymorphisms using simulated reads from a small eukaryote genome. *BMC Bioinformatics* 16**,** 382. doi:

Van Der Auwera, G.A., Carneiro, M.O., Hartl, C., Poplin, R., Del Angel, G., Levy-Moonshine, A., Jordan, T., Shakir, K., Roazen, D., Thibault, J., Banks, E., Garimella, K.V., Altshuler, D., Gabriel, S., and Depristo, M.A. (2013). From FastQ data to high confidence variant calls: the Genome Analysis Toolkit best practices pipeline. *Curr Protoc Bioinformatics* 43**,** 11.10.11-33. doi: 10.1002/0471250953.bi1110s43
